# Proving of Farfara

**Published:** 1884-11

**Authors:** Irvin J. Lane

**Affiliations:** Sing Sing, N. Y.


### A PROVING OF FARFARA.

IRVIN J. LANE, M. D., SING SING, N. Y.

The first proving of Farfara of which I will speak was made by myself by taking the tincture prepared according to the *American Homœopathic Pharmacopœia.*

I have been in good health since I can remember (excepting intermittent fever last fall).

Age twenty-two; eyes blue; hair brown; head large; form stout; features full; habits good; bowels regular; urine normal; chest large and full.

July 8th, 1883, I began proving Farfara by taking seventy-five drops of tincture. In about two minutes I felt a dryness of the pharynx, with difficult swallowing of saliva and eructations of food.

6.30 P. M.—Took two hundred drops. Had eructations of food in a few minutes afterward.

7.30 P. M.—Took two hundred drops. Eructations of wind.

8.30 P. M.—Took two hundred drops. Eructations of wind.

July 9th.—Restless and sleepless last night after going to bed. Had to get up twice during the night to urinate. (Seldom have to get up at all.) Dreamed about murder, burglars, etc., the most of the night, which would awake me, and I would feel afraid for a short time. Arm felt numb, as if asleep, with it extended above the head.

6.30 A. M.—Took two hundred drops. Had eructations of wind afterward.

9 A. M.—Took ℥ss. Eructations tasting of medicine.

11.30 A. M.—Took ʒv. In a few minutes afterward felt sleepy, with dull aching in forehead. Alternation of paleness and flushing of the face. The smell of the medicine would cause nausea.

2 P. M.—After going in bathing had a hot sensation in the forehead and a burning sensation in the nose, with a discharge of watery mucus from the nose.

3 P. M.—Took ℥ss.

4 P. M.—Photophobia while in the sun.

8 P. M.—After eating had a gurgling of wind in the abdomen with some pain and passage of a great deal flatus before stool. Temperature, 98; pulse quick and strong.

9 P. M.—Colic-like pains in hypogastric region, which come in paroxysms, with flatulence in the abdomen and passage of a great deal flatus. Aggravated by sitting up straight or leaning back; ameliorated by leaning forward or passing flatus. Thirsty, drinking little and often.

9.15 P. M.—Took two hundred drops.

July 10th.—Slept well last night, but dreamed some.

6.30 A. M.—Took two hundred drops.

10.30 A. M.—Took two hundred drops.

12.30 P. M.—Took two hundred drops.

6.30 P. M.—Felt sleepy, with constant nausea and slight colic. Felt better while leaning forward; appetite not very good; slight coryza. Feel better in the open air. Eructations after each dose.

7.30 P. M.—Colic in hypogastric region, with sharp pain in region of pouparts ligament.

10 P. M.—The smell or even the thoughts of Farfara would cause nausea. Took two hundred drops. Sour eructations. Constant gaping and very sleepy; burning, irritating sensation in the nose; aggravated by a long inspiration through the nose; dull feeling in the forehead; get the lines crossed while driving; aching pain at times in left temple; pass a great deal light, nearly colorless, urine. Sp. gr., 10.05.

July 11th.—Sensation as of sand beneath the eye-lids in the morning on first awaking; constant irritation in left side of the nose.

6.30 A. M.—Took two hundred drops. In a few minutes there was a dry sensation in the pharynx and frequent sneezing. Urine, sp. gr., 10.27.

12.30 P. M.—Took two hundred drops. The smell or thoughts of the medicine caused nausea. I took the above dose as I was about two-thirds through eating my dinner. As soon as I took it I felt full, and did not care for any more dinner. Nausea, with a sleepy, dull feeling. Base of tongue coated white and dotted with red papilla.

6.30.—Took two hundred drops. Itching of prepuce and scrotum. Colic in hypogastric region, aggravated by standing, ameliorated by exercise or lying down. Feel sleepy.

10 P. M.—Took two hundred drops. Veins look blue.

July 12th, 6.30 A. M.—Took two hundred drops. Eructations tasting of the medicine. Itching of prepuce and scrotum. Feel drowsy and dull.

1.30 P. M.—Took two hundred drops. Get tired very easy while at work. Centre and base of tongue coated white, dotted by red, elevated papilla. Appetite good.

6.30 P. M.—Itching of scrotum, prepuce, and glans penis, with fine red eruption on glans penis.

11.30 P. M.—Took two hundred drops. Mosquitoes bit my hands quite a good deal during the evening while fishing, and it swelled up like a bee sting, with a burning, hot feeling, and would itch for a long time. Burning, hot feeling in the stomach after taking the medicine.

July 13th.—Rained yesterday. Got my feet wet last night. This morning (after exposure to wet), had a bruised, aching pain in lumbar region and hips when I got up. Worse when first moving about or standing; better after continued motion. Hands swollen from the mosquito bites last night.

8 A. M.—Took two hundred drops.

1.30 P. M.—Base of tongue coated white, with red, raised papilla. Puffy swelling of hands still continues. Bruised, aching pain in lumbar region. Passage of a great deal flatus, with rumbling of wind in the abdomen.

8 P. M.—Took two hundred drops. Got tired very easily this afternoon. Hands swollen some from the mosquito bites yet.

July 14th.—Was very restless last night.

9 A. M.—Took two hundred drops.

1.30 P. M.—Took two hundred drops.

July 15th, 11 A. M.—Itching of glans penis.

12 M.—Took two hundred drops.

7.25 P. M.—Took one ounce after supper.

7.30 P. M.—Burning sensation in the throat, with dry, constricted sensation of pharynx. Great difficulty in swallowing.

7.55 P. M.—Weakness of the extremities. Right knee gave out while walking, as if I had no power of the muscles. Aching of the muscles of the right arm, worse at the elbow. Extreme weakness of the right side. Feel quite cross.

9.30 P. M.—Sensation as of strong tobacco smoke in the throat.

July 16th.—Had an irritating sensation in the upper part of the throat about 4 A. M., causing a dry, hacking cough, aggravated by lying down or taking a deep breath.

12 M.—Took two hundred drops. Dry sensation in pharynx with difficult swallowing.

July 17th.—Dry sensation in pharynx at times during the day, with difficult empty swallowing.

9 A. M.—Took two hundred drops.

11 A. M.—Took one hundred drops. Posterior part of tongue coated yellow, dotted by raised red papilla.

July 18th, 7.30.—Took two hundred drops.

July 19th.—Posterior part of tongue dotted by red raised papilla.

11 A. M.—Took two hundred drops.

July 20th, 11 A. M.—Took two hundred drops.

July 22d, 8 A. M.—Took three hundred drops.

12 M.—Took three hundred drops. Itching of scrotum.

1 P. M.—Burning in meatus urinarius while micturating, principally in the anterior part; urine dark colored and stream very large.

10 P. M.—Took two hundred drops.

July 23d.—Did not take any Farfara during the day because the thoughts or smell of it would cause nausea.

7 P. M.—Saw black, oblong specks with white rings around them before my eyes.

July 24th, 8 A. M.—Took three hundred drops.

July 25th.—Took no more Farfara, as the thoughts of it would make me feel sick.

For about eight or ten days after I stopped taking Farfara I could not laugh, as it would cause very severe pain, as though the whole chest had been bruised.

The following cannot be classed as a strictly homœopathic proving, as it was not made as a tincture or taken in the first case while the patient was in perfect health, yet I think it gives a good idea of the action of the drug on the female sex.

December 15th, 1861.—Miss A. C., age eleven. Was in perfect health up to date. Had had her menses three times at regular periods, and they should have appeared on the 20th of December, but on the 15th she went skating on the river and broke through the ice and got wet up about to the umbilical region; before she got ashore her clothes were frozen so stiff that they had to be cut from her. She had very severe pains along the spine, so that she had to keep her bed.

January 1st, 1862.—Her menses had not appeared, so Dr. White (allopath) was called to prescribe for her. He ordered the following prescription:

Take two ounces of colts-foot root, wash it thoroughly; add half pint of gin and steep it slowly, not letting it boil; then let it stand until it gets cold; then add half pint cold gin; shake well and take in teaspoonful doses four times per day for a week; then she took a dessert-spoonful four times per day for four days.

During the time she was taking Farfara she felt sleepy night and day, so that she slept the most of the time, but during the day while she was asleep she knew what was going on; could only keep awake by sitting up and talking. Pain in the back stopped while taking it. Felt tired all the while; did not want to work, talk, or be talked to, or bothered in any way. Had a dull, stupid headache all over the head.

The second day after increasing the dose she commenced to have labor-like pains. The pains were as they have been at each labor since, viz.: When they first commenced, which was in the afternoon, they commenced across the left lumbar region at about the brim of the pelvis, passing from the left to the right side, then passing from the right side back to the left, always passing around her in front. After they got to the left side, where they started from, they would pass down and out through the vulva. Just as soon as they would pass out there would other pains commence to come in the same place and go through the same circuit, so making the round trip every three to five minutes. They continued the same for about twenty-four hours, not being free from them at any time.

As soon as the pains commenced she thought she was going to die and was very restless all the time. After about twenty-four hours the pains slowly changed in character from the sharp to an aching pain, which passed along the vagina out to the vulva, where there was a continual bruised feeling which gradually passed away. She could sleep all the time. As the pains began to pass away the menstrual flow commenced. The first day after the menses commenced there was a bruised-like pain on the left side of the abdomen, which gradually passed away.

The menses lasted five or six days, were very dark and clotted until toward the last part, when they changed to a pinkish red color and were thin and watery, passing through everything, or, as she said, they resembled the lochia after labor. Menses worse when sitting.

A leucorrhœa then commenced which was about the color of milk, a little thicker than cream, and very sticky; always worse during the first few days of her menses and when walking around. She has had very hard and tedious labors ever since, the pains being about the same as described above, and lasting from twenty-four to forty-eight hours.

February 2d, 1883.—Mrs. A. M. (same person as above). Health good. Had been pregnant about six weeks when she commenced taking Farfara to cause abortion. She prepared it as follows: Take ℥ij colts-foot and one pint brandy; steep them together for about one to two hours.

9 A. M.—She commenced by taking a tablespoonful every hour and a half for the first day. Soon after taking the first dose she was thirsty, and would drink large quantities of water at a time, and quite often. Within three hours after taking the first dose she felt very sleepy, could hardly keep awake while at work. While asleep, either during the day or night, she was conscious of what was going on, and would hear or feel even the least noise or motion.

9 P. M.—As soon as she laid down, there was a sensation as if the whole contents of the abdomen were pressing up against the contents of the thorax, causing a choking sensation, so that she could not lay down; had to lean a little forward, which caused relief.

February 3d, 6 A. M.—On getting up the face felt stiff, as if she had been crying, and her whole body was bloated, so that she could not get her clothes on. The breasts were swollen more than any other part of the body, and the nipples more than the rest of the breasts. She felt as if she had drank large quantities of water and it had filled her full; the swelling was soft and flabby, as if there was cold water beneath the skin; on pressing the surface it would leave the imprints of the fingers for about five minutes, and the skin would turn light red.

10. A. M.—She commenced passing large quantities of colorless urine, which would cause a burning sensation, as if it was scalding hot. Soon after passing so much urine she had a very intense itching sensation of the vulva, which seemed almost unbearable; at first, it seemed to be on the external surface of the vulva, and was slightly relieved by scratching, but the itching gradually extended up into the vagina, until it nearly reached the cervix. There was no eruption noticed. Applications of cold and hot water, lard, and lard and honey were made with no relief to the itching, but was aggravated by lying down and after micturition, and continued for about nine days after she stopped taking the medicine. There was a continual watery discharge from the eyes; after closing the lids they felt dry, and there was a sensation as of sand beneath them. Constant excoriating coryza. Discharge of excoriating water from the nose, which would start with a tingling sensation at the bridge of the nose, aggravated by leaning forward; then there was a sensation as if a drop of cold water would start from the upper part of the nose and run down.

Toothache beginning in a decayed tooth on right side of lower jaw and would extend all the way around; teeth felt long and sore; face felt as if she had taken a severe cold, which affected the whole face; four to five days after she commenced taking Farfara she felt confused and was very forgetful; she would lay anything down, then in two or three minutes she could not tell where she had laid it.

February 6th.—A dark cloud would appear before her eyes and she would have nausea with a cold sensation in the stomach. One morning after feeling sick she vomited a green, frothy substance, tasting bitter. After the nausea and internal cold sensation there would be an external cold sensation in the region of and about the size of the stomach. The nausea always came on in the morning, and she compared it to morning sickness. Breath was very offensive. The breasts were full of milk and felt as they had at times when she had been away from her child for a long time while nursing it, except that they were so sore that she could not bear the slightest touch. The soreness was of an aching-sore character. The milk was very watery, so that it was only slightly colored. She drew the milk from one breast, which would relieve the pain for a short time. The milk continued to stay in the breasts for six or seven weeks after she stopped taking Farfara. Had a dull, heavy headache in the forehead all the time while taking it. Arms would feel numb, as if asleep, when lying with them above the head. Cold perspiration on her hands. Legs below the knees would feel numb, as if asleep, on lying down. Twitching and jerking of the legs under the knees, which would draw them up so that the feet would be drawn up nearly to the hips—they would stay drawn up until she went to sleep or would get up and walk around. Her arms would twitch in the same way at times when she was asleep, which would flex them so as to cause her to awake. Cold perspiration on her feet and legs to her knees.

